# Optimized Treatment of Refractory Hypercholesterolemia in Patients With Atherosclerotic Cardiovascular Disease or Heterozygous Familial Hypercholesterolemia With Alirocumab (OPTIMIZE)

**DOI:** 10.3389/fcvm.2022.953040

**Published:** 2022-07-15

**Authors:** Isabella Sudano, Francois Mach, Tiziano Moccetti, Thilo Burkard, Christian Fahe, Alain Delabays, Hans Rickli, Pierre-Frédéric Keller, Jörn Dopheide, Sereina Bodenmann, Tom Fiolka, Georg Ehret, David Spirk

**Affiliations:** ^1^Department of Cardiology, University Heart Center, University Hospital Zurich, Zurich, Switzerland; ^2^Division of Cardiology, University Hospitals Geneva, Geneva, Switzerland; ^3^Cardiocentro Ticino, Lugano, Ticino, Switzerland; ^4^Department of Cardiology, University Hospital Basel, Basel, Switzerland; ^5^Praxis Fahe AG, Muhen, Muhen, Switzerland; ^6^Service of Cardiology, Hospital Morges, Morges, Switzerland; ^7^Clinic of Cardiology, Cantonal Hospital St. Gallen, St Gallen, Switzerland; ^8^Cardiology Center Delemont, Delemont, Switzerland; ^9^Clinic of Angiology, Cantonal Hospital Chur, Chur, Switzerland; ^10^Faculty of Medicine, University of Bern, Bern, Switzerland; ^11^Medical Department, Sanofi, Vernier, Switzerland; ^12^Institute of Pharmacology, University of Bern, Bern, Switzerland

**Keywords:** atherosclerotic cardiovascular disease, heterozygous familial hypercholesterolemia, LDL-C target attainment, PCSK9 inhibition, statin intolerance

## Abstract

**Background:**

Low-density lipoprotein cholesterol (LDL-C) is a major risk factor for atherosclerotic cardiovascular disease (ASCVD). In confirmatory trials, proprotein convertase subtilisin/kexin type 9 inhibitor alirocumab substantially lowered LDL-C and reduced cardiovascular morbidity and mortality. However, the routine clinical use of alirocumab in Switzerland has not yet been studied.

**Methods:**

In this prospective nation-wide cohort study, we aimed to investigate the patient profile and routine clinical efficacy and safety of alirocumab in 207 patients with ASCVD or heterozygous familial hypercholesterolemia and increased LDL-C despite maximally tolerated statin therapy. LDL-C was measured at baseline and after 3-months follow-up.

**Results:**

Overall, mean age was 63 ± 11 years, 138 (67%) were men, and 168 (81%) had statin intolerance (SI). Patients with SI had a higher baseline LDL-C (4.3 ± 1.4 vs. 3.3 ± 1.4 mmol/l; *p* < 0.001) and less frequently ASCVD (71% vs. 95%; *p* = 0.002). After 3 months of treatment with alirocumab, LDL-C was reduced from 4.1 ± 1.5 to 2.0 ± 1.2 mmol/l (50.5%; *p* < 0.001). Mean absolute and relative reductions in LDL-C were similar in patients with vs. without SI (2.2 ± 1.2 vs. 1.9 ± 1.3 mmol/l; *p* = 0.24 and 49.0 vs. 56.6%; *p* = 0.11, respectively). In total, adverse events were recorded in 25 (12%) patients, with no new safety signals.

**Conclusions:**

In routine clinical practice, alirocumab was predominantly used in patients with SI suggesting that the great majority of patients with insufficient LDL-C control who would be candidates for alirocumab are not receiving this therapeutic option in Switzerland. LDL-C lowering was potent and similar in patients with and without SI, replicating the favorable efficacy-safety profile of alirocumab from randomized trials.

## Introduction

Atherosclerotic cardiovascular disease (ASCVD) remains the most frequent cause of death in the Western world. Approximately a third of all deaths can be attributed to cardiovascular diseases, half thereof to coronary heart disease (CHD) (1). Hypercholesterolemia is a main risk factor for the development of atherosclerosis and therewith of ASCVD. Although several potent lipid-lowering therapies are available, many patients with familial hypercholesterolemia (FH) and non-familial hypercholesterolemia continue to have increased low-density lipoprotein cholesterol (LDL-C) levels (2) and thus remain at high risk for the occurrence or reoccurrence of adverse vascular events (3).

The inhibition of proprotein convertase subtilisin/kexin type 9 (PCSK9) *via* monoclonal antibodies (mAb), is a recent approach offering an additional treatment option for high-or very high-risk patients with hypercholesterolemia not reaching the LDL-C targets despite oral treatment. Alirocumab is a fully human mAb that binds specifically to PCSK9, leading to a decrease in LDL-C *via* increased LDL-receptor activity. This compound was studied in the large clinical phase III study program ODYSSEY, comprising of 22 individual trials with more than 24,500 patients. A substantial and sustained LDL-C reduction of 50 or 60% was observed in these studies for alirocumab 75 or 150 mg every 2 weeks, respectively, with no serious safety issues as compared to placebo (4). In the largest trial of this program, the ODYSSEY OUTCOMES study, treatment with alirocumab significantly reduced the rate of major adverse cardiovascular events (MACE) and was associated with a lower mortality of any cause in almost 19,000 patients after acute coronary syndrome (5).

In a mutual consensus statement of the European Society of Cardiology (ESC) and European Atherosclerosis Society (EAS) from 2017, the expert panel recommended considering PCSK9 inhibitor (PCSK9i) mAbs in very high risk patients with ASCVD and substantially elevated LDL-C despite maximum tolerated statin treatment with or without ezetimibe, and in high or very high risk patients with heterozygous FH (heFH) in primary prevention and substantially increased LDL-C levels despite maximum tolerated statin plus ezetimibe treatment (6).

According to the 2019 ESC/EAS Guidelines for the Management of Dyslipidaemias, treatment with PCSK9 inhibitors is recommended in patients with ASCVD (grade IA) or heFH with another major risk factor (grade IC) who do not achieve their goal on a maximum tolerated dose of statin and ezetimibe (7). In Switzerland, the reimbursement criteria for PCSK9i mAbs are defined by the Federal Office of Public Health (FOPH) and since 2019, include LDL-C >2.6 in patients with ASCVD and LDL-C >4.5 or >5.0 mmol/l in heFH patients with or without another major risk factor, respectively (8).

In the present prospective Swiss national-wide cohort study, we aimed to investigate the patient profile, efficacy and safety of alirocumab in patients with ASCVD and/or heFH and markedly increased LDL-C despite maximally tolerated statin therapy with or without other lipid-lowering medication used and reimbursed under routine clinical conditions.

## Materials and Methods

### Study Population

In the *Prospective non-interventional study for the optimized treatment of refractory hypercholesterolemia in patients with heterozygous familial hypercholesterolemia or clinically apparent atherosclerotic cardiovascular disease with alirocumab (OPTIMIZE)* prospective multi-center national cohort study, hospital- and private-practice based cardiologists, neurologists, angiologists, diabetologists, nephrologists, and selected FOPH-approved lipid specialists across all geographic regions of Switzerland were invited to include patients during a 15-months period between April 2019 and June 2020. For the purpose of representativeness, Switzerland was divided into five geographic sectors of similar territorial and population size, and for each of these sectors, 10 physicians were randomly selected from a centralized national register and asked to participate in this study.

Inclusion criteria were age ≥18 years, ASCVD and/or heFH, and increased LDL-C despite maximally tolerated statin therapy with or without other lipid-lowering medication in accordance with the Swiss SmPC (4) and the Swiss reimbursement criteria for alirocumab (8). These criteria included LDL-C >2.6 and >3.5 mmol/l in ASCVD patients with and without rapid progression, as well as >4.5 and >5.0 mmol/l in heFH patients with and without additional risk factors, respectively. All patients received subcutaneous injections of alirocumab 75 or 150 mg every 2 weeks. Patients were already prescribed, or the intention to prescribe alirocumab was made at the discretion of the treating physician, prior to enrollment in our study. Exclusion criteria were current participation in any interventional study, ongoing treatment with lipid apheresis, contraindication for the use of alirocumab according to the Swiss SmPC (4), and inability to provide informed consent.

The study period included a 12-week follow-up after the baseline visit. The primary endpoint was the mean percent reduction in LDL-C after 12 weeks of lipid-lowering treatment including alirocumab.

### Data and Definitions

Data were collected by treating physicians and entered in a standardized case report form. At baseline, following parameters were documented: Patient characteristics including age, gender, height, weight, and blood pressure, family history of cardiovascular disease, diagnosis of heFH according to the Dutch Lipid Clinic Network (DLCN) criteria (9), acute and chronic co-morbidities including hypertension, diabetes mellitus (DM), smoking status, ASCVD including CHD, stroke, and peripheral arterial disease, and renal impairment, LDL-C target value determined in accordance with then contemporary consensus statement guidelines, laboratory values including lipids, liver enzymes, blood sugar, and renal parameters, diet, lifestyle measures, and lipid lowering treatment within prior 12 months, date of start and dosage of treatment with alirocumab, presence and detailed description of statin intolerance, and the occurrence of adverse events. LDL-C was calculated using the Friedewald formula (10). At follow-up, patient characteristics including weight and blood pressure, laboratory values including lipids, liver enzymes, blood sugar, and renal parameters, current lipid lowering treatment, change (if any) and current dosage of treatment with alirocumab, and the occurrence of adverse events were recorded.

The diagnosis of ASCVD included the following: Acute coronary syndrome (myocardial infarction or unstable angina), stable angina pectoris, stroke, transitory ischemic attack, peripheral arterial disease, infrarenal aortic aneurysm, and a history of coronary or other arterial revascularisation procedure. The diagnosis of heFH was based on the DLCN criteria as recommended by the consensus statement of the EAS on the diagnosis and treatment of familial hypercholesterolaemia (9). A “definite or probable diagnosis of FH” could be made if the total subject score reached >6 points. Of note, genotypic information was only determined for a small proportion of patients, hence the patients identified as heFH were technically “patients with FH.” For simplicity of reading and regulatory matters we retain the term heFH throughout this manuscript.

Statin intolerance (SI) was considered proven if (i) therapeutic attempts with several statins resulted in myalgia, or (ii) an increase of creatinine kinase levels to at least five times the upper limit of normal was observed, or (iii) severe statin-related hepatopathy occurred, in accordance with the Swiss reimbursement criteria for PCSK9 inhibitors (8). For the purpose of the present analysis, patients were separated into two groups: those with and without documented statin intolerance.

The LDL-C target attainment was assessed according to the 2016 and 2019 ESC/EAS guidelines on the management of dyslipidemias (7, 11), both of which were adopted by the Working Group on Lipids and Atherosclerosis of the Swiss Society of Cardiology. For the assessment of LDL-C target attainment, both 2016 and 2019 guidelines were used to account for recommendations of the revised LDL-C targets released in September 2019, thus during the course of the study. Based on the 2016 version of the guidelines, the LDL-C target was <1.8 mmol/l AND at least 50% reduction if baseline LDL-C was between 1.8 and 3.5 mmol/l in patients with ASCVD or DM with target organ damage or a major risk factor, and it was <2.6 mmol/l AND at least 50% reduction if baseline LDL-C was between 2.6 and 5.2 mmol/l in patients with markedly elevated single risk factors (e.g., FH or severe arterial hypertension) or DM without target organ damage or a major risk factor without ASCVD (11). Based on the 2019 version of the guidelines, the LDL-C target was <1.4 mmol/l AND at least 50% reduction from baseline LDL-C in patients with ASCVD, DM with target organ damage, or FH with another major risk factor, and it was <1.8 mmol/l AND at least 50% reduction from baseline LDL-C in patients with markedly elevated single risk factors (e.g., severe hypercholesterolemia or severe arterial hypertension), DM without target organ damage, or FH patients without ASCVD and without another major risk factor (7). Responders were defined as individuals with at least 40% reduction in LDL-C from baseline, in accordance with the Swiss reimbursement criteria for PCSK9 inhibitors (8).

### Statistical Analysis

Based on previous phase III studies with alirocumab, we assumed a near to normal distribution for the change in LDL-C and a standard deviation (SD) of 25%. For an estimated mean reduction in LDL-C of 40%, the necessary sample size was 200 patients to reach a precision of 6.93% for the length of the two-sided 95% confidence interval (CI), translating into a corresponding 95% CI of [36.54%; 43.47%].

Continuous variables with a normal distribution are presented as means with standard deviations, and group comparisons were performed using a *t*-test; continuous variables with skewed distribution are described as median values with interquartile ranges (IQR), and group comparisons were performed using a rank-sum test. Discrete variables are depicted as frequencies and percentages, and group comparisons were performed with the chi square or Fisher's exact test. Data were analyzed using the SAS statistical software (version 9.4).

## Results

### Patient Characteristics

Overall, 207 patients were enrolled by 44 participating specialist centers. Mean age was 63 ± 11 (range 36–83) years, 89 (43%) patients were elderly (≥65 years of age) and 138 (67%) male (Table 1). Mean baseline LDL-C was 4.1 ± 1.5 mmol/l, TC of 6.1 ± 1.6 mmol/l, HDL-C of 1.5 ± 1.0 mmol/l, TG of 1.9 ± 1.1 mmol/l, and Lp (a) of 63 ± 70 mg/dl.

**Table 1:** Demographics, comorbidities, and lipid-lowering medication according to the presence of statin intolerance.

	**SI (*****N*** **=** **168)**		**No SI (*****N*** **=** **39)**		**Total (*****N*** **=** **207)**		***P***
**Demographics**							
Age, mean years ± SD	63	10	60	12	63	11	0.09
Elderly (age ≥65 years), *n* (%)	76	(45.2)	13	(33.3)	89	(43.0)	0.18
Women, *n* (%)	59	(35.1)	10	(25.6)	69	(33.3)	0.26
Systolic BP, mm Hg ± SD	135	17	132	19	134	17	0.49
Diastolic BP, mm Hg ± SD	80	10	78	12	80	10	0.28
**Comorbidities**							
Manifest ASCVD, *n* (%)	120	(71.4)	37	(94.9)	157	(75.8)	0.002
Revascularization, *n* (%)	86	(71.7)	26	(70.3)	112	(71.3)	0.87
MI, *n* (%)	53	(44.2)	24	(64.9)	77	(49.0)	0.028
Unstable AP, *n* (%)	17	(14.2)	7	(18.9)	24	(15.3)	0.48
PAD, *n* (%)	13	(10.8)	4	(10.8)	17	(10.8)	0.99
Stroke, *n* (%)	10	(8.3)	3	(8.1)	13	(8.3)	0.97
TIA, *n* (%)	8	(6.7)	2	(5.4)	10	(6.4)	0.78
Aortic aneurysm	2	(1.7)	2	(5.4)	4	(2.5)	0.21
HeFH, *n* (%)	38	(22.6)	17	(43.6)	55	(26.6)	0.008
Obesity (BMI ≥30 kg/m^2^), *n* (%)	34	(20.2)	14	(35.9)	48	(23.2)	0.037
Diabetes mellitus, *n* (%)	27	(16.1)	6	(15.4)	33	(15.9)	0.92
**Lipid-Lowering Medication**							
Statin, *n* (%)	51	(30.4)	37	(97.4)	88	(42.5)	<0.001
Ezetimibe, *n* (%)	52	(31.0)	31	(81.6)	83	(40.1)	<0.001
Alirocumab 75 mg, *n* (%)	112	(66.7)	30	(76.9)	142	(68.6)	0.29
Alirocumab 150 mg, *n* (%)	52	(31.0)	9	(23.1)	61	(29.5)	0.29

*SI, statin intolerance; SD, standard deviation; BP, blood pressure; ASCVD, atherosclerotic cardiovascular disease; MI, myocardial infarction; AP, angina pectoris; TIA, transient ischaemic attack; PAD, peripheral arterial disease; HeFH, heterozygous familial hypercholesterolemia; BMI, body mass index*.

At baseline, 157 (76%) patients had manifest ASCVD, 55 (27%) heFH (confirmed by genetic diagnostics in 10 cases), 33 (16%) diabetes mellitus (type 2 in 32 cases), and 168 (81%) statin intolerance. Among the 157 patients with ASCVD, 112 (71%) had a history of coronary or other arterial revascularisation procedure, 77 (49%) myocardial infarction, 24 (15%) unstable angina pectoris, 17 (11%) peripheral arterial disease, 13 (8%) stroke, 10 (6%) transient ischaemic attack, and 4 (3%) aortic aneurysm.

Among the 168 patients with SI at baseline, the reason for SI diagnosis was myalgia under several statins in 159 (95%) patients, increase in creatinine kinase in 13 (8%), and severe hepatopathy in 1 (1%). Patients with SI had less often ASCVD (71% vs. 95%; *p* = 0.002) and heFH (23% vs. 44%; *p* = 0.008) than those without SI, no other differences in anthropometrics between the two groups were noted (Table 1). Mean baseline LDL-C and TC were higher, and Lp (a) was lower in patients with vs. without SI (Table 2).

**Table 2:** Baseline laboratory parameters according to the presence of statin intolerance.

	**SI (*****N*** **=** **168)**		**No SI (*****N*** **=** **39)**		**Total (*****N*** **=** **207)**		***P***
LDL-C, mean mmol/l ± SD	4.3	1.4	3.3	1.4	4.1	1.5	<0.001
TC, mean mmol/l ± SD	6.4	1.6	5.2	1.5	6.1	1.6	<0.001
HDL-C, mean mmol/l ± SD	1.5	1.1	1.4	0.7	1.5	1.0	0.38
TG, mean mmol/l ± SD	1.9	1.1	1.9	1.3	1.9	1.1	0.99
Lp (a), mean mg/dl ± SD	46	57	100	84	63	70	0.02
eGFR, mean ml/min/1.73 m^2^ ± SD	77	20	80	19	77	20	0.35

*SI, statin intolerance; SD, standard deviation; LDL-C, low-density lipoprotein cholesterol; TC, total cholesterol; HDL-C, high-density lipoprotein cholesterol; TG, triglycerides; Lp (a), lipoprotein (a); eGFR, estimated glomerular filtration rate*.

### LDL-C Treatment

During the 12-months period prior to the baseline visit, 124 (60%) patients were treated with atorvastatin (median dose 40 mg), 116 (56%) rosuvastatin (20 mg), 34 (16%) pitavastatin (2 mg), 27 (13%) simvastatin (40 mg), and 128 (62%) with ezetimibe (10 mg). In comparison to patients with SI, those without SI more frequently used pitavastatin (19% vs. 5%), simvastatin (15% vs. 5%), and ezetimibe (72% vs. 60%), respectively. Patients without SI received a higher median dose of atorvastatin (80 vs. 40 mg), rosuvastatin (20 vs. 10 mg), and pitavastatin (4 vs. 2 mg) and a lower dose of simvastatin (30 vs. 40 mg) than those with SI.

Sixty nine percent of patients were initiated on alirocumab 75 mg and 30% on alirocumab 150 mg every 2 weeks, the dose was unknown in 1% (Table 1). Patients with and without SI were similarly often treated with alirocumab 75 mg (67% and 77%; *p* = 0.29) and 150 mg (31% and 23%). The injection of alirocumab was administered into the abdominal wall in 42% of patients, the thigh in 41%, and the upper arm in 7%, the site of administration was unknown in 10%. In total, concomitant statin therapy was used in 43% of patients and ezetimibe in 40%, and their use was more frequent in patients without SI (Table 1).

### LDL-C Reduction, LDL-C Target Attainment, and Tolerability

**Figure 1:**
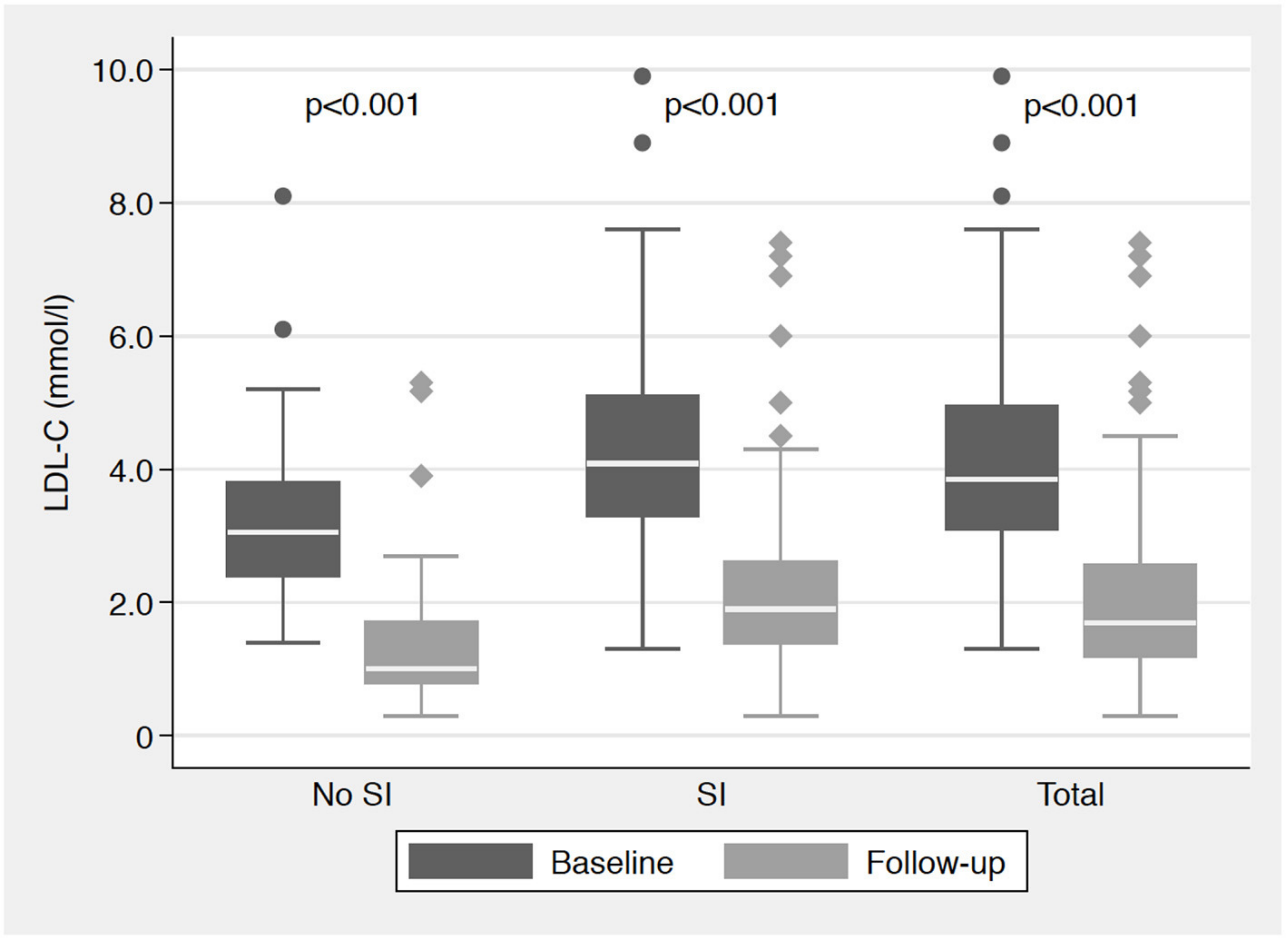
Median (IQR) LDL-C (mmol/l) at baseline and follow-up visit according to the presence of statin intolerance. IQR, interquartile range; LDL-C, low-density lipoprotein cholesterol; SI, statin intolerance; all *p*-values for the comparisons of LDL-C at follow-up vs. baseline.

After 3 months, mean LDL-C was reduced from 4.1 ± 1.5 to 2.0 ± 1.2 mmol/l (*p* < 0.001); in patients with SI from 4.3 ± 1.4 to 2.1 ± 1.2 mmol/l (*p* < 0.001), and in those without SI from 3.3 ± 1.4 to 1.4 ± 1.2 mmol/l (*p* < 0.001). Mean absolute and relative reductions in LDL-C were 2.2 ± 1.2 mmol/l and 50.5%; and both were similar in patients with vs. without SI (2.2 ± 1.2 vs. 1.9 ± 1.3 mmol/l; *p* = 0.24 and 49.0 vs. 56.6%; *p* = 0.11, respectively). The development of the median (IQR) LDL-C is displayed in Figure 1. There was a trend toward a higher mean absolute reduction in LDL-C for patients treated with alirocumab 150 vs. 75 mg every 2 weeks (2.4 ± 1.3 vs. 2.0 ± 1.2 mmol/l; *p* = 0.11).

Overall, mean absolute (relative) reduction in TC was 2.2 ± 1.4 mmol/l (33.5%), mean HDL-C increase 0 ± 0.1 mmol/l (5.1%), and mean TG reduction 0.2 ± 1.0 mmol/l (3.0%).

**Figure 2:**
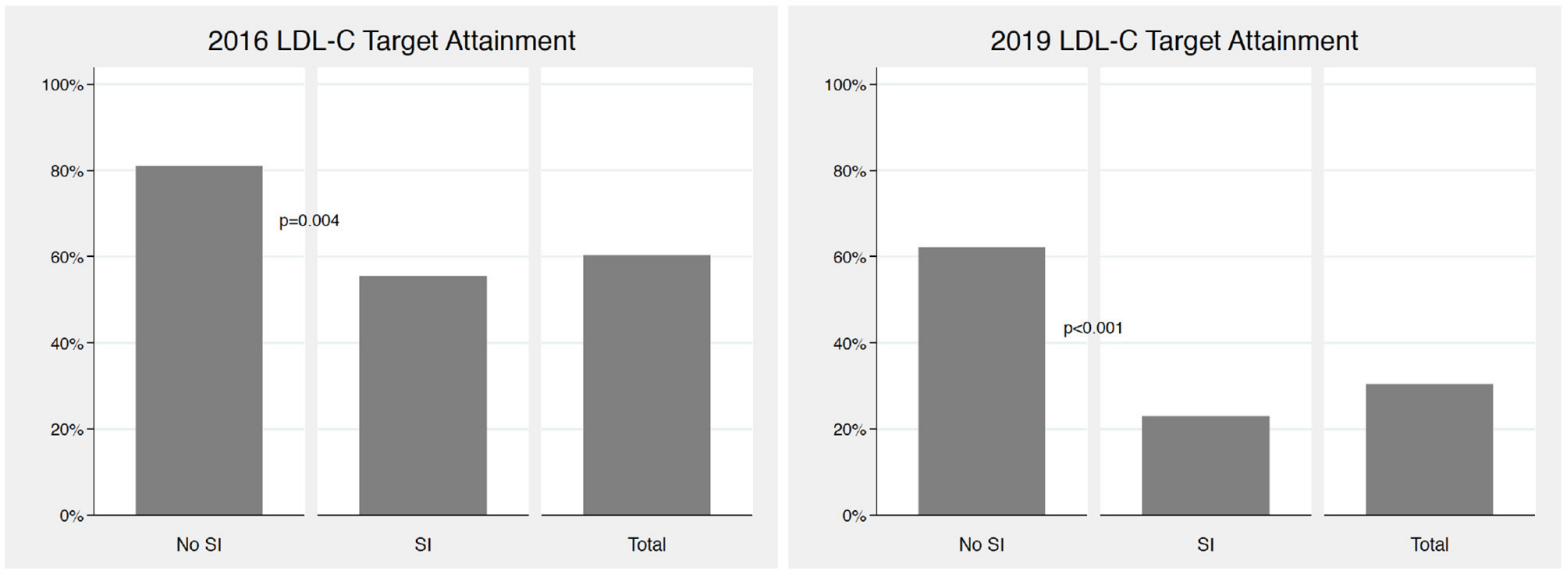
2016 and 2019 ESC/EAS guidelines LDL-C target attainment according to the presence of statin intolerance. LDL-C, low-density lipoprotein cholesterol; ESC, European Society of Cardiology; EAS, European Atherosclerosis Society; SI, statin intolerance; both *p*-values for the comparisons of LDL-C target attainment in patients with vs. without SI.

The 2016 ESC/EAS guidelines-recommended targets for LDL-C were reached in 60% (95% CI 53–67%) patients; they were attained in 55% (95% CI 48–63%) patients with and 81% (95% CI 68–94%) without SI (p=0.004). The 2019 ESC/EAS guidelines-recommended targets for LDL-C were reached in 30% (95% CI 24–37%) patients; they were achieved in 23% (95% CI 16–30%) patients with and 62% (95% CI 46–79%) without SI (*p* < 0.001). The 2016 and 2019 guidelines-recommended target attainments are depicted in Figure 2.

According to the Swiss reimbursement criteria for PCSK9 inhibitors, the proportion of responders was practically identical between patients with and without SI (76% vs. 75%; *p* = 0.95). According to the LDL-C targets set by physicians at baseline visit, the response rate was 50% (95% CI 43–57%); it was 44% (95% CI 36–52%) in patients with and 76% (95% CI 62–90%) without SI (*p* < 0.001).

During the study period, adverse events were recorded in 25 (12%) patients and were classified as serious in 5 (2.4%) patients requiring hospitalization. The most frequent adverse events included flu-like symptoms in 8 (3.9%) patients, local injection-site reactions in 7 (3.4%), angina pectoris in 6 (2.9%), and myalgia in 4 (1.9%). Some patients had more than one adverse event. Alirocumab was discontinued in 6 (2.9%) cases. There was no difference in the incidence of adverse events between patients with and without SI, except for myalgia which occurred in the group with SI only (Table 3). Also, the rate of adverse events was similar between patients treated with alirocumab 75 and 150 mg every 2 weeks (12.7 and 11.5%; *p* = 0.81).

**Table 3:** Adverse events according to the presence of statin intolerance.

	**SI (*****N*** **=** **168)**		**No SI (*****N*** **=** **39)**		**Total (*****N*** **=** **207)**		***P***
Any adverse event, *n* (%)	20	(11.9)	5	(12.8)	25	(12.1)	0.87
Serious adverse event, *n* (%)	3	(1.8)	2	(5.1)	5	(2.4)	0.22
Flu-like symptoms, *n* (%)	7	(4.2)	1	(2.6)	8	(3.9)	0.64
Injection-site reaction, *n* (%)	6	(3.6)	1	(2.6)	7	(3.4)	0.75
Angina pectoris, *n* (%)	5	(3.0)	1	(2.6)	6	(2.9)	0.89
Myalgia, *n* (%)	4	(2.4)	0	(0.0)	4	(1.9)	0.33

*SI, statin intolerance*.

## Discussion

In our prospective observational cohort study conducted in 44 specialist centers across Switzerland, alirocumab was predominantly used in patients with SI. Mean reductions in LDL-C after 3-month treatment with alirocumab in routine clinical practice replicated the favorable results from confirmatory trials, and they were similar between patients with and without SI. Close to two thirds of patients treated with alirocumab plus statin reached the guideline-recommended targets for LDL-C. In contrast, despite a high response rate to treatment with alirocumab, more than three quarters of patients with SI did not reach LDL-C targets. Alirocumab may be considered an effective and safe treatment option in very high-risk patients not reaching LDL-C targets on oral lipid-lowering therapies. OPTIMIZE is one of the largest real-world evidence studies with alirocumab to date, and importantly, the large proportion of SI patients suggests that the great majority of high-risk patients with insufficient LDL-C control who would be candidates for PCSK9 inhibition are not receiving this therapeutic option.

Our results observed in a routine clinical setting confirm the beneficial effect and a very good safety profile of alirocumab established by randomized controlled trials in high-risk patients both with and without SI. In patients with SI, the relative LDL-C reduction at 3 months from baseline was almost identical in the OPTIMIZE (49%) and the ODYSSEY ALTERNATIVE (47%) studies while baseline LDL-C was somewhat higher in the latter one (4.3 and 5.0 mmol/l, respectively) (2). Due to the increase in PCSK9 production following administration of statins through upregulation of sterol regulatory element binding protein-2, a synergistic effect of PCSK9 inhibition plus HMG-CoA reductase inhibition and thus potentiation of LDL-C reduction appears to be feasible in patients without SI (12, 13). Indeed, such tendency was described in the early phase III studies from the ODYSSEY development program of alirocumab; the 12-weeks LDL-C reduction was 52% in the COMBO II and 47% in the ALTERNATIVE studies (14). This indirect comparison should be interpreted with caution. However, a similar trend toward a higher efficacy of alirocumab in patients without vs. with SI was observed in our study (57% vs. 49%, respectively). In contrast, patients with SI derived a higher absolute clinical benefit regarding MACE from treatment with alirocumab than those without SI in the OUTCOMES study, due to the higher baseline LDL-C and consequently, the higher baseline risk of adverse outcome (15). Of note, the observed effect on LDL-C in our study is attributable solely to treatment with alirocumab because patients needed to be on a stable concomitant lipid-lowering therapy within 3 months prior to the initiation of alirocumab and no change of such therapy was mandated, in accordance with the Swiss reimbursement criteria for PCSK9 inhibitors (8).

Along the same lines, the proportion of patients reaching the 2016 ESC/EAS guidelines-recommended LDL-C targets was remarkably similar between confirmatory trials with alirocumab and our observational study (11). Namely, these targets were attained in 78% patients on treatment at week 24 in the ODYSSEY COMBO I study and 81% patients without SI from OPTIMIZE, and they were reached in 51% patients on treatment at week 24 in the ODYSSEY ALTERNATIVE study and 55% patients with SI from OPTIMIZE. These results are reassuring because in randomized controlled trials, patients invariably are (i) selected and (ii) regularly and tightly monitored whereas in real-world studies, patients tend to more often have (i) co-morbidities and poly-medication and (ii) a lower compliance. Not surprisingly, the rate of target attainment was lower in patients with vs. without SI in both confirmatory and observational settings, as a consequence of the higher baseline LDL-C in those who cannot be treated with statins.

Our results also complement the landscape of other real-world studies conducted outside Switzerland confirming a strong overall efficacy of alirocumab. In the non-interventional German PEARL study, 619 patients with high baseline LDL-C levels were enrolled across 345 study centers. As a result, a reduction of LDL-C of 49% against baseline (least squares mean) after 24 weeks was reached in combination with a favorable safety profile (16). Additionally, in the related Austrian PEARL-AT study, a mean relative reduction of LDL-C levels by 50% was reported in a cohort group of 112 patients at 24 weeks (17). Similar results have also been reported from clinical practice in the UK with significant LDL-C reductions by 54% at 4 months after alirocumab initiation in a retrospective study including 150 patients (18). Furthermore, in a study from Netherlands including 106 patients receiving alirocumab, mean LDL-C reductions above 55% were attained. However, it should also be noted that for 121 patients who were prescribed evolocumab, no substantial differences in LDL-C lowering were found compared to alirocumab (19). Moreover, in a real-world cohort consisting of 271 patients treated with PCSK-9 inhibitors (evolocumab and alirocumab) in the USA, a median LDL-C lowering for alirocumab 75 mg of 59% after 12 months was reported. Again, both PCSK9i mAbs showed very similar outcomes regarding efficacy and safety (20).

Besides the high efficacy and favorable safety profile in all of the existing real-world studies, a high proportion of patients on PCSK9 inhibitors with at least partial SI (between 42 and 82%) was previously observed (16–20). In fact, a high prevalence of SI in both real-world scenarios and randomized controlled trials has been described and in great detail reviewed in the literature of over the past decade, and represents a significant therapeutic challenge in clinical practice (21, 22). Statin-associated muscle symptoms (SAMS) were reported in 7 to 29% of all statin users and represent the main reason for non-adherence, often leading to discontinuation of treatment (23). As a consequence of suboptimal adherence or interruption of the treatment, patients are exposed to a significantly higher risk of ASCVD (24). Moreover, SAMS are sometimes difficult to diagnose and assess objectively, and their accurate frequency is still uncertain (25). Specifically, the reported incidence for myopathy is commonly below 5% in randomized controlled trials since patients with known SI are often excluded from the study already during the screening period, causing a potential selection bias (26). The high proportion of SI in our study is likely due to the difficulties of many patients in tolerating oral lipid-lowering medications and the physician desire for improved control of strongly elevated LDL-C levels to obtain the highest absolute clinical benefit. Statin intolerant patients were reported to have significantly less muscle symptoms with alirocumab but a considerable proportion of SI patients remained symptomatic in a previous trial (2), an observation that we did not confirm in our study. Importantly, the beneficial potential of advanced lipid-lowering options such as alirocumab is greatly underused when the routine clinical prescription is largely limited to statin intolerant patients, as suggested by the high percentage of SI patients in our trial.

In alignment with the ODYSSEY OUTCOMES study allowing for the dose up-titration of alirocumab from 75 to 150 mg every 2 weeks, the majority of patients in OPTIMIZE were treated with alirocumab 75 mg (5). Likely because many of the patients with alirocumab 150 mg were statin intolerant in our study, we observed only a trend toward a higher efficacy of this dose, possibly owing to the absence of the synergistic effect of PCSK9 inhibition and statin treatment. However, there was no difference in the beneficial safety profile of the two doses, in line with the ODYSSEY phase III development program (4).

Our study has several limitations: First, the study was observational and the follow-up was limited to a short-term period of 12 weeks only. Thus, no assessment of long-term efficacy and safety of alirocumab in a real-world setting was possible. Second, the choice of lipid-lowering therapy was not determined by an experimental plan but solely as a result of the treatment decision by physicians in charge. Third, we cannot exclude considerable inclusion bias due to (i) the reimbursement criteria for PCSK9 inhibitors imposed by the regulators of the Swiss health care system and (ii) because participating physicians were asked to enroll up to 10 patients only and may have selected those who were likely to respond best, e.g., those with the highest LDL-C at baseline or those untreated due to SI. Due to lack of randomization and lack of a comparison arm in our study, the results are to be considered hypothesis generating, not confirmatory, and must therefore be interpreted with caution. In contrast to randomized controlled trials, our study had no exclusion criteria and the results reflect the contemporary clinical practice. Finally, the study was conducted in Switzerland only and the results may not be generalizable to other countries or regions.

In conclusion, the OPTIMIZE study confirms the favorable efficacy and safety profile of alirocumab in routine clinical setting as compared to randomized controlled trials among high risk patients with hypercholesterolemia despite maximally tolerated statin therapy. In Switzerland, alirocumab was predominantly used in patients with SI and very high LDL-C in whom a large room for improvement regarding guideline-recommended target attainment persists. Alirocumab may be considered an effective and safe treatment option in very high-risk patients not reaching LDL-C targets on oral lipid-lowering therapies, both with and without SI. The large proportion of SI patients in our study suggests that many high-risk patients with insufficient LDL-C control, who are numerically more frequent, are not offered PCSK9 inhibition as a therapeutic option, representing a loss to optimal preventive cardiovascular therapy.

## Data Availability Statement

Qualified researchers may request access to patient-level data and related documents (including, e.g., the clinical study report, study protocol with any amendments, blank case report form, statistical analysis plan, and dataset specifications). Patient-level data will be anonymized, and study documents will be redacted to protect the privacy of trial participants. Further details on Sanofi's data sharing criteria, eligible studies, and process for requesting access can be found at https://www.vivli.org/.

## Ethics Statement

The study was approved by the leading ethics committee of the canton Zurich and by the ethics committees of all participating centres in accordance with local regulations. All participants signed a written informed consent, and the study was conducted in agreement with the declaration of Helsinki.

## Author Contributions

IS, SB, and DS were responsible for the study conception and design. IS, FM, TM, TB, CF, AD, HR, P-FK, JD, and GE substantially contributed to the data acquisition. IS, TF, and DS were involved in the data interpretation. IS and DS drafted the manuscript. FM, TM, TB, CF, AD, HR, P-FK, JD, SB, TF, and GE critically revised the manuscript for intellectual content. All authors approved the final version of the manuscript.

## Funding

The study was funded by Sanofi, Vernier, Switzerland. Data collection, data management, and analyses were conducted by an independent clinical research organization.

## Conflict of Interest

IS received speaker fee and research support by Sanofi as well as travel and personal fees by Amgen, Astra Zeneca, Daiichi Sankyo, Menarini, MSD, Recordati, and Servier. TB received research funding to institution by CSEM, Collabree, Medtronic, Roche Diagnostics, and Sanofi as well as travel, speaker, or advisory board fees by Boehringer Ingelheim, Daiichi Sankyo, Novartis, Sanofi, and Servier. SB, TF, and DS are employees of Sanofi, Vernier, Switzerland, and may hold shares and/or stock options in Sanofi. GE received grants or consulting fees to institution from Amgen, Novartis, Sanofi, and Servier, and personal royalties or licenses from UpToDate. No other conflict of interest was reported from the authors regarding the content of this manuscript.

## Publisher's Note

All claims expressed in this article are solely those of the authors and do not necessarily represent those of their affiliated organizations, or those of the publisher, the editors and the reviewers. Any product that may be evaluated in this article, or claim that may be made by its manufacturer, is not guaranteed or endorsed by the publisher.
